# Supplementary material for: Mechanism for fluctuating pair density wave
Source: Nat Commun. 2023 Jun 1;14:3181. doi: 10.1038/s41467-023-38956-x (PMC10235120; doi:10.1038/s41467-023-38956-x)
Supplement: Supplementary file 1 — Supplementary Information [file 41467_2023_38956_MOESM1_ESM.pdf]

Supplementary Information

Chandan Setty, Laura Fanfarillo, and P. J. Hirschfeld
(Dated: May 7, 2023)

SUPPLEMENTARY NOTE 1: DERIVATION OF THE EFFECTIVE ACTION FOR THE FLUCTUATING COOPER PAIRS

We consider a single band superconducting (SC) system, assuming a parabolic band dispersion $\xi_{\mathbf{k}} = \epsilon_{\mathbf{k}} - \mu$ where $\epsilon_{\mathbf{k}} = \mathbf{k}^2/2m$ and μ is the chemical potential. The pairing interaction is given by

$$H_I = -g \sum_{\mathbf{q}} \theta^{\dagger}_{\mathbf{q}} \theta_{\mathbf{q}}, \quad (1)$$

$$\theta_{\mathbf{q}} = \sum_{\mathbf{k}} f_{\mathbf{k},\mathbf{q}}\, c_{-\mathbf{k}+\frac{\mathbf{q}}{2},\downarrow} c_{\mathbf{k}+\frac{\mathbf{q}}{2},\uparrow} \quad (2)$$

g is the constant SC coupling, $f_{\mathbf{k},\mathbf{q}}$ is the angular factor defined as $f_{\mathbf{k},\mathbf{q}} = (h_{\mathbf{k}-\mathbf{q}/2} + h_{\mathbf{k}+\mathbf{q}/2})/2$, with $h_{\mathbf{k}} = \left(k_x^2 - k_y^2\right)/\Lambda$, where Λ is the pairing energy cut-off.

We can decouple the interaction term using the standard Hubbard-Stratonovich (HS) transformation [1]. The resulting action is given by

$$S = \sum_{k,\sigma} c^{\dagger}_{k\sigma}(-i\omega_n + \xi_{\mathbf{k}}) c_{k\sigma} + \sum_q \frac{|\Delta_q|^2}{g} - \sum_q \left[\Delta^*_q \theta_q + \theta^{\dagger}_q \Delta_q\right] \quad (3)$$

where Δ_q is the HS field associated with θ_q and we put $k = (\mathbf{k}, \omega_n)$ and $q = (\mathbf{q}, \Omega_m)$. Introducing the Nambu spinor, $\psi^{\dagger}_k = (c_{k,\uparrow} \;\; c_{-k,\downarrow})$ we can rewrite the action as

$$S = \sum_q \frac{|\Delta_q|^2}{g} + \sum_{kk'} \psi^{\dagger}_{k'} A_{kk'} \psi_k \quad (4)$$

with the $A_{kk'}$ matrix defined as

$$\mathrm{diag}(A_{kk'}) = [(-i\omega_n + \xi_{\mathbf{k}})\delta_{kk'}, (-i\omega_n - \xi_{\mathbf{k}})\delta_{kk'}]$$

$$A_{kk'}|_{12} = (A_{kk'}|_{21})^* = -f_{\mathbf{k},\mathbf{k}-\mathbf{k}'} \Delta_{k-k'}$$

We can integrate out the fermions and separate $A_{kk'} = -G_0^{-1} + \Sigma_{kk'}$. G_0^{-1} contains the contribution of the homogeneous and constant components of the Hubbard-Stratonovich field (i.e. $q = 0$) while the $\Sigma_{kk'}$ contains the fluctuating part. Thus the action reads

$$\begin{aligned} S &= \sum_q \frac{|\Delta_{\mathbf{q}}|^2}{g} - \mathrm{Tr} \ln[-G_0^{-1} + \Sigma_{kk'}] \\ &= \sum_q \frac{|\Delta_{\mathbf{q}}|^2}{g} - \mathrm{Tr} \ln[G_0^{-1}] + \sum_n \frac{1}{n} \mathrm{Tr}[(G_0 \Sigma)^n]. \end{aligned} \quad (5)$$

Here

$$G_0(k) = \begin{pmatrix} \dfrac{-i\omega_n - \xi_{\mathbf{k}}}{\omega_n^2 + E_{\mathbf{k}}^2} & \dfrac{f_{\mathbf{k},0}\Delta_0}{\omega_n^2 + E_{\mathbf{k}}^2} \\ \dfrac{f_{\mathbf{k},0}\Delta_0}{\omega_n^2 + E_{\mathbf{k}}^2} & \dfrac{-i\omega_n + \xi_{\mathbf{k}}}{\omega_n^2 + E_{\mathbf{k}}^2} \end{pmatrix} = \begin{pmatrix} \mathcal{G}_0(k) & \mathcal{F}_0(k) \\ \mathcal{F}_0(k) & -\mathcal{G}_0(-k) \end{pmatrix} \quad (6)$$

with $E_{\mathbf{k}} = \xi_{\mathbf{k}}^2 + f_{\mathbf{k},0}^2 \Delta_0^2$; the fluctuation contributions of the pairing field is contained instead in

$$\Sigma(k - k') = \begin{pmatrix} 0 & -f_{\mathbf{k},\mathbf{k}-\mathbf{k}'} \Delta_{k-k'} \\ -f_{\mathbf{k},\mathbf{k}-\mathbf{k}'} \Delta^*_{k'-k} & 0 \end{pmatrix} \quad (7)$$

The saddle-point equation $\partial S / \partial \Delta_0 = 0$ explicitly reads

$$\frac{2\Delta_0}{g} = \mathrm{Tr}[G_0(k) \frac{\partial G_0^{-1}(k)}{\partial \Delta_0}] \quad (8)$$

evaluating the trace the above expression reduces to $g^{-1} - f_{\mathbf{k},0}\mathcal{F}_0(k) = 0$, i.e. explicitly

$$g^{-1} + \frac{T}{V} \sum_k \frac{f_{\mathbf{k},0}^2}{(i\omega_n + E_{\mathbf{k}})(i\omega_n - E_{\mathbf{k}})} = 0, \quad (9)$$

the usual BCS equation. Eq.(9) has to be solved self-consistently with the equation for the chemical potential.

To analyze the Gaussian fluctuations of SC field around the mean field solution, we have to consider the expansion of Eq. (5) up to the second order. Given the complex nature of the HS field we can rewrite the fluctuating term in Eq.(7) as $\Sigma(q) = -f_{\mathbf{k},\mathbf{q}}[Re\Delta_q \sigma_1 + Im\Delta_q \sigma_2]$, where $\sigma_{1/2}$ are Pauli matrices. The Gaussian action reads

$$S_G = \sum_q \eta^T_{-q} \begin{pmatrix} \dfrac{1}{g} + \Pi^{11}_q & \Pi^{12}_q \\ \Pi^{21}_q & \dfrac{1}{g} + \Pi^{22}_q \end{pmatrix} \eta_q. \quad (10)$$

Here $\eta^T_q = (Re\Delta_q, Im\Delta_q)$ and Π^{ij}_q are the particle-particle propagator components

$$\Pi^{ij}_q = \frac{T}{V} \sum_k Tr[G_0(k+q)\sigma_i G_0(k) \sigma_j] f^2_{\mathbf{k},\mathbf{q}} \quad (11)$$

Using Eq.(6) in Eq. (11) one can write explicitly the element Π_q^{ij}. We refer the reader to Appendix A of Ref. [2] for a detailed derivation, here we only report and discuss the results relevant to our study. The diagonal terms $\Pi_q^{11/22}$, control the Gaussian fluctuation $|Re\Delta|^2$ and $|Im\Delta|^2$ and are proportional to the antisymmetric ad symmetric combination of the normal and anomalous Green functions. The off-diagonal term, Π_q^{12}, couples real and imaginary part of the fluctuating field and is proportional to the convolution of the normal Green function only. As discussed in [2] it can be shown that $\Pi^{12}(q) \sim \Omega_m \mathcal{O}(q^2)$ so that it vanishes in the static limit, leading to the effective decoupling between the amplitude and phase fluctuations.

In this work we want to investigate the emergence of a spatially modulated pairing fluctuation out of a homogeneous d-wave SC state. To do that, we consider the static limit (in which fluctuation of $Re\Delta$ and $Im\Delta$ are decoupled) and focus explicitly of the amplitude mode only. Thus, in what follows, we only consider the Π_q^{11} element of the particle-particle propagator defined in Eq. (11)

$$\Pi_q = -\frac{T}{V}\sum_k [\mathcal{G}_0(k+q)\mathcal{G}_0(-k) - \mathcal{F}_0(k+q)\mathcal{F}_0(k)] f^2_{\mathbf{k},\mathbf{q}}. \tag{12}$$

where we simplify the notation as $\Pi_q^{11} \to \Pi_q$, and analyze the Gaussian action, Eq. 10, that reduces to

$$S_G = \sum_q L_q^{-1}|\Delta_q|^2 = \sum_q [g^{-1} + \Pi_q]|\Delta_q|^2 \tag{13}$$

It is worth noticing that above T_i, where $\Delta_0 = 0$ and the anomalous Green functions vanish, the propagator Π_q^{ij}, Eq.(11), becomes fully diagonal and proportional to the identity, i.e. it is not longer possible to distinguish between fluctuations of the real and imaginary part of the pairing field. In this case, the particle propagator reduces to $\Pi_q \sim -\mathcal{G}_0(k+q)\mathcal{G}_0(-k) f^2_{\mathbf{k},\mathbf{q}}$. Notice that, even in the normal phase, where the SC gap is zero, the anisotropy of the pairing affects the momentum dependence of the propagator due to the overall form factor $f^2_{\mathbf{k},\mathbf{q}}$.

SUPPLEMENTARY NOTE 2: MOMENTUM DEPENDENCE OF THE PAIRING SUSCEPTIBILITY

By expanding the Gaussian action, Eq.(13) at small frequency and momentum up to second order, we obtain the familiar expression

$$S_G = \sum_q (c_0 + c_2\mathbf{q}^2 + \gamma|\Omega_m|)|\Delta_q|^2 \tag{14}$$

where $c_0 = g^{-1} + \Pi_0 = 0$ is the mass term that vanishes at the instability temperature, c_2 describes the momentum rigidity of the fluctuating Cooper pairs, and γ is the damping coefficient that at weak coupling reduces to the microscopic value of the Ginzburg-Landau damping $\gamma = \pi N_F/8T$, where N_F is the density of states at the Fermi level [3]. As we mentioned above, in this work we are interested in the analysis of static spatially modulated SC state, thus we stady the static limit and analyze the momentum dependency only.

In the above analysis we implicitly assumed that the broken phase is a static homogeneous superconductor, i.e. we assumed that the minimum of the action is associated with the homogeneous and constant value of the order parameter Δ_0, Eqs. (8) and (9). This implies that the highest temperature for which the pairing susceptibility $L_{\mathbf{q}}^{-1}$ diverges is determined by the mass term c_0 of Eq.(14). Notice, however that, if the highest temperature associated with the divergence of $L_{\mathbf{q}}^{-1}$ is found at for finite $\mathbf{q} = \mathbf{Q}$, this means that the instability is associated with a spatially non-uniform SC mode. If this is the case, it means that the finite momentum fluctuations are actually lowering the energy in Eq.(13).

In this work we use a perturbative approach to explore the possible emergence of a spatially modulated SC state from the homogeneous d-wave superconductor. We study the momentum dependence of the static pairing susceptibility of the homogeneous d-wave superconductor and look for a negative sign of the momentum rigidity parameter c_2 in Eq.(14). Notice that, in order to analyze the stability of the modulated SC phases, we will need to go beyond the second order in the momentum expansion

$$L_{\mathbf{q}}^{-1} = \sum_n c_n \mathbf{q}^n, \quad \text{with} \quad c_n = \frac{1}{n}\frac{\partial^n L_{\mathbf{q}}^{-1}}{\partial \mathbf{q}^n}\bigg|_{\mathbf{q}=0} \tag{15}$$

In the following we assume $2m = 1$, as a consequence energies have dimensions of 2-D V^{-1}, and $L_{\mathbf{q}}^{-1}$ is therefore dimensionless.

SUPPLEMENTARY NOTE 3: NUMERICAL ANALYSIS

We analyze the mean-field solution at finite temperature as a function of the pairing strength $\alpha = E_B/E_F$. We explore the space of the parameters at low-density n fixing the Fermi energy $E_F = n/N_F$, where $N_F = m/2\pi$ is the density of states at the Fermi level in 2D, and varying the superconducting coupling g in Eq.(9). For simplicity we use the weak-coupling relation between the bound state energy and the SC coupling, i.e. $E_B = \Lambda e^{-1/N_F g}$. The mean field results for Δ and μ as a function of temperature for different values of the pairing strength α are shown in Fig.2 of the main text.

To study the fluctuations around the mean-field values of Δ we analyze the static pairing susceptibility, $L_{\mathbf{q}}^{-1} = g^{-1} + \Pi_{\mathbf{q}}$, defined in Eqs. (12)- (14), by setting

$\Omega_m = 0$. Using the explicit expression for the normal and anomalous Green functions, Eq. (6), the pairing propagator of Eq.(12) reads

$$\Pi_{\mathbf{q}} = \frac{T}{V}\sum_{\mathbf{k},n} \frac{(i\omega_n + \xi_{\mathbf{k+q}})(i\omega_n - \xi_{\mathbf{k}}) - f_{\mathbf{k},0} f_{\mathbf{k+q},0}\Delta^2}{(\omega_n^2 + E_{\mathbf{k}}^2)(\omega_n^2 + E_{\mathbf{k+q}}^2)} f^2_{\mathbf{k},\mathbf{q}} \quad (16)$$

that can be evaluated at each temperature by using for $\Delta(T)$ and $\mu(T)$ the mean-field values obtained solving the self-consistent equations discussed in the previous section. After performing the Matsubara summation we can write the particle-particle propagator as

$$\Pi_{\mathbf{q}} = \frac{1}{V}\sum_{\mathbf{k}} \mathcal{P}_{\mathbf{q}}(\mathbf{k}) \quad (17)$$

where we defined $\mathcal{P}_{\mathbf{q}}(\mathbf{k})$ as

$$\begin{aligned} \mathcal{P}_{\mathbf{q}}(\mathbf{k},\omega_n) = & \frac{f^2_{\mathbf{k},\mathbf{q}}}{2E_{\mathbf{k+q}}E_{\mathbf{k}}} \Bigg\{ \frac{n_F(E_{\mathbf{k+q}}) - n_F(E_{\mathbf{k}})}{E_{\mathbf{k+q}} - E_{\mathbf{k}}} \times \\ & \big(E_{\mathbf{k+q}}E_{\mathbf{k}} - \xi_{\mathbf{k+q}}\xi_{\mathbf{k}} - f_{\mathbf{k+q},0} f_{\mathbf{k},0}\Delta^2\big) + \\ & - \frac{n_F(E_{\mathbf{k+q}}) - n_F(-E_{\mathbf{k}})}{E_{\mathbf{k+q}} + E_{\mathbf{k}}} \times \\ & \big(E_{\mathbf{k+q}}E_{\mathbf{k}} + \xi_{\mathbf{k+q}}\xi_{\mathbf{k}} + f_{\mathbf{k+q},0} f_{\mathbf{k},0}\Delta^2\big) \Bigg\} \end{aligned} \quad (18)$$

and n_F is the Fermi function. To obtain the polynomial form discussed in Eq. (15), we expand the integrand $\mathcal{P}_{\mathbf{q}}(\mathbf{k})$ around $\mathbf{q} = 0$. We fix $\mathbf{q}$ along x, but the expansion along different $\mathbf{q}$ directions can generally yield quantitatively distinct results due to the anisotropy. Hereafter our derivatives are meant as ∂q_x. The mass terms is given by

$$c_0 = g^{-1} + \frac{1}{V}\sum_{\mathbf{k}} \mathcal{P}_{\mathbf{q}}(\mathbf{k})|_{\mathbf{q}=0} \quad (19)$$

while the higher order coefficients read

$$c_n = \frac{1}{V}\sum_{\mathbf{k}} I_n(\mathbf{k}) \quad \text{with} \quad I_n(\mathbf{k}) = \frac{\partial \mathcal{P}_{\mathbf{q}}(\mathbf{k})}{\partial \mathbf{q}^n}\bigg|_{\mathbf{q}=0} \quad (20)$$

The momentum dependence of $I_2(\mathbf{k})$ at $T = 0$ and at T_i is shown in Fig.5 of the main text where we report both the s-wave and d-wave case.

For the set of parameters used in this work we need to expand up to sixth order to check the stability of the finite momentum state. In Fig. 2 of the main text we show the behavior of c_n, $n = 2, 4, 6$ as a function of α at T_i. We use dimensionless units i.e. renormalized c_n as $c_n\Lambda^{n/2}$. In Supplementary Fig. 1, we report the results at $T = 0$. As one can see also at zero temperature we find

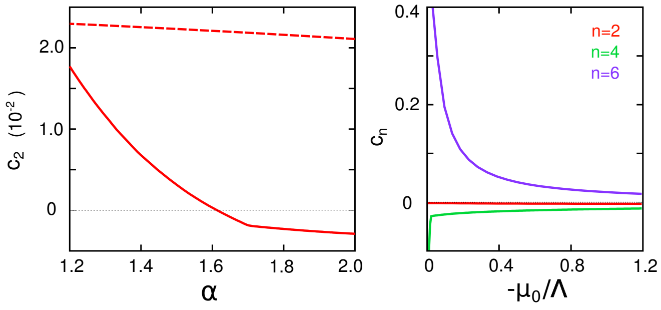

Supplementary Figure 1: (color online) Coefficients of the q-expansion at $T = 0$. (a) For the anisotropic d-wave interactions $c_2(\alpha)$ (solid line) changes sign for $\alpha \sim 1.6$ that correspond to a sign change of the chemical potential. No sign change is found within the range of parameters studied for the isotropic s-wave case (dashed line). (b) Higher order coefficients c_4, c_6 for the d-wave case vs μ_0. We shown only the $\mu_0 < 0$ region for which $c_2 < 0$.

a sign change of the momentum rigidity parameter c_2 at strong coupling where we also find $c_4 < 0$ and $c_6 > 0$. The critical value of the interaction at which the rigidity vanishes is slightly larger $\alpha \sim 1.6$ than the one found at the instability temperature. Notice that in terms of μ we find again that the sign change of c_2 occur in the same range of interactions in which μ changes its value from positive to negative.

In addition to T_i and $T = 0$, we numerically compute $c_2(\alpha)$ at finite temperature for $T < T_i$ to build the phase diagram in Fig.1 of the main text where we explicitly show the critical temperature $T^*(\alpha)$ along which the rigidity parameter vanishes.

SUPPLEMENTARY NOTE 4: CHARACTERIZATION OF THE MODULATED SC PHASE

We can derive information about the modulated SC phase analyzing the static susceptibility written as a sixth degree polynomial

$$L^{-1}_{\mathbf{q}} \sim c_0 + c_2\mathbf{q}^2 + c_4\mathbf{q}^4 + c_6\mathbf{q}^6 \quad (21)$$

where the odd terms vanish for symmetry. The extrema of $L^{-1}(\mathbf{q})$ occur at $\mathbf{q} = \pm Q_\pm$ with

$$Q_\pm = \Big(\frac{-c_4 \pm \sqrt{c_4^2 - 3c_2c_6}}{3c_4}\Big)^{1/2} \quad (22)$$

Let's assume that the minima occur at finite momentum, in this case one can further expand the polynomial around $\mathbf{Q}$ so that we can find an approximate expression for the susceptibility of the modulated SC phase

$$L^{-1}_{\mathbf{q}} \sim \bar{c_0} + \bar{c_2}(\mathbf{q} - \mathbf{Q})^2 \quad (23)$$

with $\bar{c_0}$ and $\bar{c_2}$ the mass and the momentum rigidity associated with the modulated SC phase and defined in terms

of the c_n coefficients and the momentum characterizing the PDW phase $\mathbf{Q}$ as

$$\begin{aligned}\bar{c_0} &= L_{\mathbf{q}}^{-1}|_{\mathbf{q}=\mathbf{Q}} = c_0 + c_2|\mathbf{Q}|^2 + c_4|\mathbf{Q}|^4 + c_6|\mathbf{Q}|^6 \\ \bar{c_2} &= \frac{1}{2}\frac{\partial L_{\mathbf{q}}^{-1}}{\partial \mathbf{q}^2}\bigg|_{\mathbf{q}=\mathbf{Q}} = c_2 + 6c_4|\mathbf{Q}|^2 + 15c_6|\mathbf{Q}|^4\end{aligned} \tag{24}$$

It is worth noticing that by assuming for the mass term of the homogeneous state a standard Ginzburg-Landau temperature dependence , $c_0 = a(T - T_i)$ with $a > 0$, we can easily recover an analogous expression for $\bar{c_0}$

$$\bar{c_0} = a(T - \bar{T_i}) \quad \text{with} \quad \bar{T_i} = T_i + \delta T \tag{25}$$

where $\delta T = \delta T = \frac{1}{a}(c_2|\mathbf{Q}|^2 + c_4|\mathbf{Q}|^4 + c_6|\mathbf{Q}|^6)$. For the set of parameter used in this work we verify that when $c_2 < 0$ this correction is positive, i.e. the instability temperature associated with the PDW phase is higher than the one of the d-wave homogeneous one.

Analogously one can verify that in the region of the phase diagram defined by $c_2 < 0$ the momentum rigidity parameter $\bar{c_2}$ associated with the PDW state is now positive, meaning that the divergence of the susceptibility occurs at $\bar{T_i}$ and is determined indeed by $\bar{c_0} = 0$, while any momentum fluctuation around $\mathbf{Q}$ increases the energy of the system.

For example, given a pairing interaction $\alpha = 0.72$, we find at T_i that the critical fluctuations at finite momentum are characterized by a wave-vector $|\mathbf{Q}| \sim 0.9\sqrt{\Lambda}$ and a positive momentum rigidity parameter $\bar{c_2}$. The correlation length can be derived from $\bar{c_2}$ as $\xi = \sqrt{\bar{c_2}/N_F}$. For the set of parameter used here we find that at T_i ξ is of the same order of magnitude of the length scale set by the energy cut-off of pairing, i.e. $\xi \sim 0.65/\sqrt{\Lambda}$.

SUPPLEMENTARY NOTE 5: NON-UNIVERSALITY OF CRITICAL α

In the numerical analysis shown in the manuscript we that $c_2(\alpha)$ changes sign for a critical value of the coupling strenght both at $T = 0$ and $T = T_i$. It is worth noticing that the critical value of α is a finite non-zero number that does not take a universal value. This is in fact affected by our choice of parameters, e.g. the filling or the cut-off energy Λ. We provide an example in Supplementary Fig. 2 where we show the behavior of $c_2(\alpha)$ for different interaction cut-offs Λ.

As one can see the values of α for which c_2 changes sign decreases as the cut-off energy parameter is increased but finite. For $\Lambda = 14$, the critical *alpha* is ~ 0.3 which is well within the weak coupling BCS regime showing the non-universality of the boundary of the phase diagram.

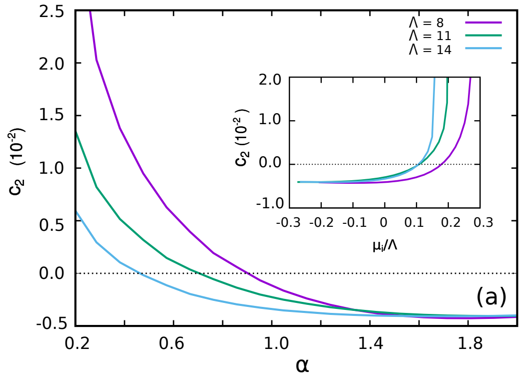

Supplementary Figure 2: Momentum rigidity parameter as a function of the coupling strength $\alpha = E_B/E_F$ at $T = T_i$ computed using different values of the cut-off energy Λ. The critical value of α for which c_2 changes signs decreases for larger values of the cut-off energy parameter.

SUPPLEMENTARY NOTE 6: ANALYTICAL DERIVATION OF THE SIGN CHANGE OF THE MOMENTUM RIGIDITY PARAMETER

In this work we analyze numerically the momentum dependence of the static pairing susceptibility, and show that for intermediate value of the interaction the SC fluctuations at finite momentum become critical. We use as a proxy the sign change of the momentum rigidity parameter c_2 that becomes negative at a critical interaction α, whose exact value depends on temperature and system parameters. In this section, we derive an approximate formula that provides evidence of the sign change of the quadratic coefficient of the d-wave SC susceptibility, although it gives a quantitatively imprecise value of the chemical potential where it actually occurs in the fully self-consistent evaluation.

We focus on the analysis of the SC fluctuation at the instability temperature, $T \to T_i^+$. The particle-particle propagator Eq. (12) in this case reads

$$\Pi(\mathbf{q}, \Omega_k) = -T\sum_{\mathbf{k}n} \mathcal{G}_0(\mathbf{k}+\mathbf{q}, \omega_n + \Omega_k)\mathcal{G}_0(-\mathbf{k}, -\omega_n), \tag{26}$$

and as we are interested in the static case we set $\Omega_k = 0$.

The $\mathcal{O}(q^0)$ term of the SC susceptibility at T_i reduces to

$$g^{-1} + T\sum_{\mathbf{k},n} \frac{f_{\mathbf{k},0}^2}{(i\omega_n - \xi_{\mathbf{k}})(i\omega_n + \xi_{\mathbf{k}})} = 0. \tag{27}$$

in agreement with Eqs. (16)-(19) once we set $\Delta = 0$. By solving it together with

$$E_F = T\ln\left(1 + e^{\mu/T}\right) \tag{28}$$

for the chemical potential at T_i, we derive the expression

for T_i and $\mu(T_i)$. This yields for $8\pi/g < 1$

$$\frac{\mu_i}{\Lambda} = \frac{1}{2}\left(8\pi g^{-1} - 1\right) \quad (29)$$

$$T_i = \frac{|\mu_i|}{\ln\left(\frac{|\mu_i|}{E_F}\right)} \quad (30)$$

where μ_i is the chemical potential at $T = T_i$. To obtain the contribution from $\mathcal{O}(q^2)$ terms, we need to evaluate $\partial^2\Pi_{\mathbf{q}}/\partial\mathbf{q}^2$:

$$\Pi_{\mathbf{q}}^{(2)} = \frac{Tq^2\Lambda^{-2}}{(2\pi)^2}\sum_{n=-N}^{N} \times \int_0^\Lambda \frac{4\pi k^3 dk(i\omega_n+\mu)(k^2+i\omega_n+\mu)}{(k^2-i\omega_n-\mu)^3(k^2+i\omega_n-\mu)} \quad (31)$$

To see that the sign change occurs for a negative μ, first set $\mu = 0$. We obtain

$$-\Pi_{\mathbf{q}}^{(2)}|_{\mu=0} = \frac{q^2\kappa}{(2\pi)^2(\kappa^2+\Lambda^2)} > 0 \quad (32)$$

with $\kappa = (2N+1)\pi$. Note $-\Pi^{(2)}(\mathbf{q},0)|_{\mu=0} \to 0$ as $N \to \infty$. To obtain the opposite sign, the finiteness of Λ has to be taken into account while allowing for the Matsubara frequency to be large. An approximate trend with μ can be obtained as

$$-\Pi_{\mathbf{q}}^{(2)}|_{\Lambda<\infty} \sim \frac{-q^2}{4\pi}\sum_{n=-N}^{N}\frac{1}{(2n+1)^2\pi^2+\mu^2} < 0 \quad (33)$$

Hence there must be a critical value of the chemical potential for which the coefficient must change sign. This is given for finite N and Λ as

$$\bar{\mu}_i^* \simeq -\frac{\bar{\Lambda}_i^2\psi'(N)}{\pi^2\ln N} \quad (34)$$

where $\psi'(N)$ is the first derivative of the digamma function, $\bar{\mu}_i = \frac{\mu_i}{T_i}$ and $\bar{\Lambda}_i = \frac{\Lambda}{T_i}$. Note the order in which N and Λ go to infinity matters. That is, $\bar{\mu}_i^* \to 0$ for $N \to \infty$ before $\bar{\Lambda}_i$. Otherwise, we see that $\bar{\mu}_i^* \to \infty$. Hence the presence of a lattice is an important ingredient in the problem.

An analogous conclusion can be arrived at zero temperature with an anisotropic d-wave gap function. The expansion is already tedious at second order but can nevertheless be evaluated as a function of the chemical potential. A similar sign change of occurs at a critical value of the zero temperature μ. The full self-consistent calculation has been performed via numerical analysis and it is shown in the main text.

[1] J. Negele and H. Orland, *Quantum Many Particle System* (Addison-Wesley, 1988).

[2] M. Marciani, L. Fanfarillo, C. Castellani, and L. Benfatto, Phys. Rev. B **88**, 214508 (2013).

[3] A. Larkin and A. Varlamov, *Theory of fluctuations in superconductors* (Clarendon Press, 2005).
